# Magnetic State Control of Non-van der Waals 2D Materials by Hydrogenation

**DOI:** 10.1021/acs.nanolett.3c04777

**Published:** 2024-03-06

**Authors:** Tom Barnowsky, Stefano Curtarolo, Arkady V. Krasheninnikov, Thomas Heine, Rico Friedrich

**Affiliations:** † Theoretical Chemistry, Technische Universität Dresden, Dresden 01062, Germany; ‡ Institute of Ion Beam Physics and Materials Research, Helmholtz-Zentrum Dresden-Rossendorf, Dresden 01328, Germany; § Center for Extreme Materials, Duke University, Durham, North Carolina 27708, United States; ∥ Materials Science, Electrical Engineering, and Physics, Duke University, Durham, North Carolina 27708, United States; ⊥ Center for Advanced Systems Understanding (CASUS), Helmholtz-Zentrum Dresden-Rossendorf, Görlitz 02826, Germany

**Keywords:** 2D materials, non-van der Waals compounds, passivation, magnetism, data-driven research, computational materials science, high-throughput computing

## Abstract

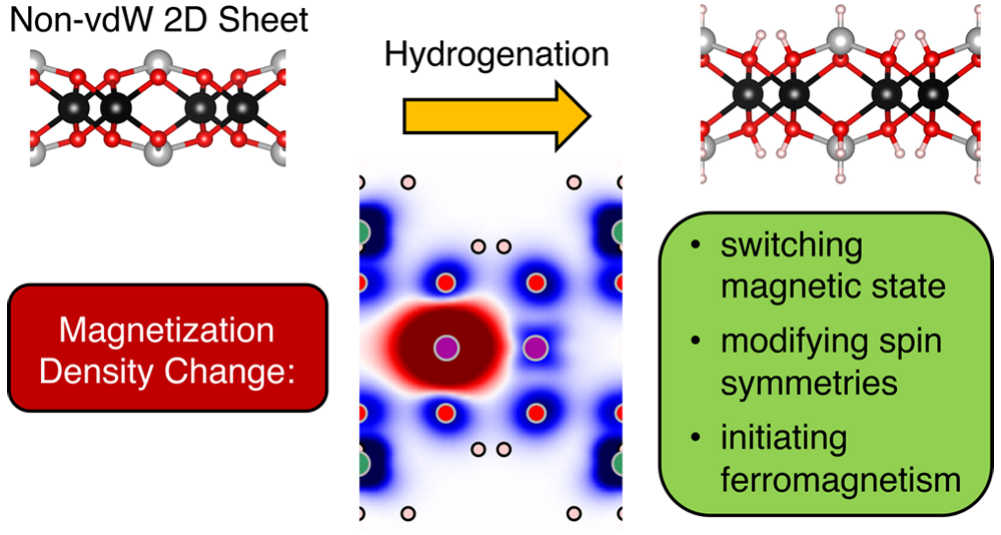

Controlling the magnetic state of two-dimensional (2D) materials is crucial for spintronics. By employing data-mining and autonomous density functional theory calculations, we demonstrate the switching of magnetic properties of 2D non-van der Waals materials upon hydrogen passivation. The magnetic configurations are tuned to states with flipped and enhanced moments. For 2D CdTiO_3_—a diamagnetic compound in the pristine case—we observe an onset of ferromagnetism upon hydrogenation. Further investigation of the magnetization density of the pristine and passivated systems provides a detailed analysis of modified local spin symmetries and the emergence of ferromagnetism. Our results indicate that selective surface passivation is a powerful tool for tailoring magnetic properties of nanomaterials, such as non-vdW 2D compounds.

Two-dimensional (2D) materials—traditionally deduced from bulk layered compounds bonded by weak van der Waals (vdW) forces—are emerging as an appealing platform to study fundamental magnetic interactions as well as for spintronic applications. Ferromagnets were identified for single layers of Cr_2_Ge_2_Te_6_^1^ and CrI_3_^2^ with Curie temperatures up to 45 K beating the Mermin–Wagner theorem due to anisotropy.^3^ Magnetic phase transitions above room temperature were observed for monolayers of VSe_2_,^4^ Fe_3_GeTe_2_,^5^ and MnSe_2_.^6^

Control over the magnetic properties has been demonstrated via different routes utilizing, for instance, pressure,^7,8^ electrostatic doping,^9^ or electric fields.^10−12^ These magnetic 2D materials are thus promising for spintronics since they can be naturally incorporated into planar device geometries which already led to first demonstration of large magnetoresistance ratios.^13−15^ For such traditional 2D systems, achieving direct control over the magnetic state via surface functionalization is, however, challenging due to their high chemical stability.

The novel class of non-vdW 2D materials^16,17^ derived from nonlayered crystals with strong ionic or covalent bonds offers qualitatively new opportunities based on the engineering of their reactive surfaces. Several magnetic representatives such as 2D hematene^18^ and ilmenene^19^ are derived from natural ores of transition metal oxides hematite (α-Fe_2_O_3_) and ilmenite (FeTiO_3_). These are complemented by various other compounds identified in experiments^20−38^ and several dozens of systems suggested by recent data-driven investigations.^39−41^ While these materials are also studied for their catalytic,^18,19^ electrochemical,^24^ and optoelectronic^42^ properties, the magnetic features related to potential room temperature ferromagnetism and spin canting are attracting particular attention.^18,19,34,43−47^

These 2D sheets have been outlined to exhibit (magnetic) cations at their surface^18,19,39^ making them appealingly susceptible to environmental influences to control their magnetic state. Since broken bonds emerge upon exfoliation of the non-vdW 2D systems from the bulk parent compound, chemical control of the magnetic properties due to surface passivation is a promising approach. As already speculated by Kaur and Coleman,^17^ such a surface passivation could be selectively used to create “activated” interfaces with desired functionalities.

Here, we show by data mining and autonomous density functional theory calculations that the magnetic state of non-vdW 2D systems can be controlled by hydrogenation. Out of an ensemble of pristine candidates, four systems exhibit a net energy gain and also pass stability tests related to supercell reconstructions and phonon mode analysis. The magnetic configuration can be switched from ferrimagnetic to antiferromagnetic or to a state with enhanced moments due to hydrogen coverage. For nonmagnetic 2D CdTiO_3_, the emergence of ferromagnetism upon passivation is observed which can be traced back to a change of the transition metal oxidation states. The findings are underscored by a visualization of the magnetization density difference, which clearly indicates the breaking of local spin symmetries and the onset of ferromagnetism.

## Passivation Types

**Figure 1:**
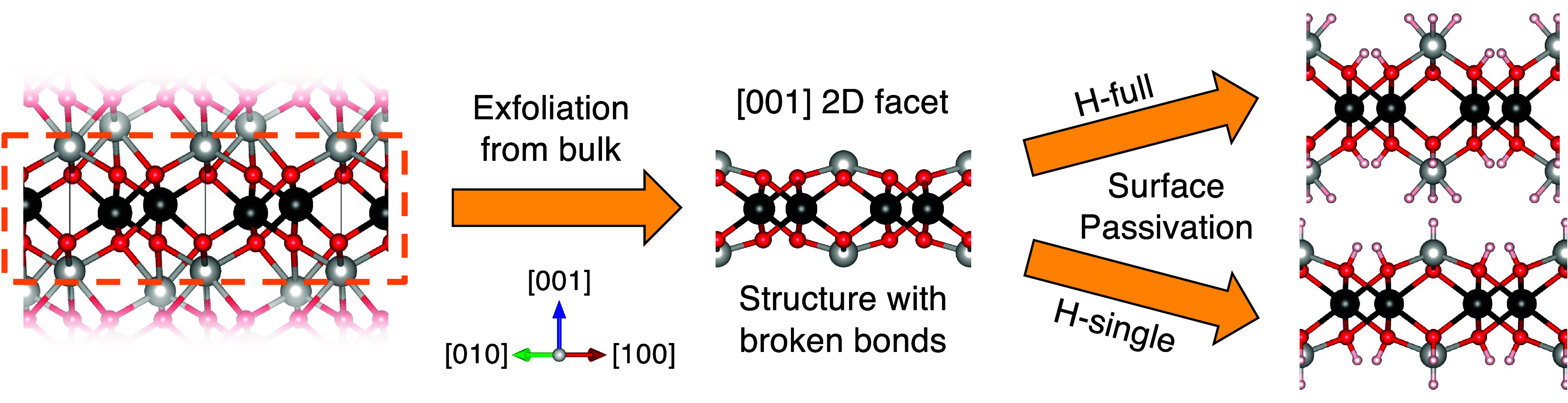
From exfoliation to passivation. Exfoliation of the 2D facets (orange frame) perpendicular to the [001] direction from the bulk crystal^18,19,39^ leads to broken bonds to be saturated by two different surface passivation types. The compass indicates the crystal directions for all structures. Colors: cations, light gray and black; anion, red; H, off-white.

We consider the combined set of 35 previously suggested non-vdW 2D materials^39,40^ excluding hematene for which computational surface passivation has already been studied in detail.^48^ The set contains 32 oxides, 2 sulfides, and 1 chloride. Note that these systems are derived from the same crystal prototype (binaries: space group #167, Pearson symbol hR10, Wyckoff positions c;e and ternaries: space group #148, Pearson symbol hR10, Wyckoff positions c;c;f). The calculations are carried out with the AFLOW software^49,50^ according to the computational details described in the Methods section. Here, hydrogen passivation is investigated. For graphene, it has been reported that reversible hydrogenation can drastically change the electronic properties by transforming the system from the metallic state into insulating graphane.^51,52^ The most intuitive approach is to passivate all (cation–anion) bonds broken upon exfoliation of the slab from the bulk (Figure 1, upper path). This corresponds to one H at each of the anions (O^2–^, S^2–^, or Cl^–^) and three at each surface metal cation.^48^ This passivation type, referred to as “H-full”, includes 12 H atoms (6 H_2_ molecules) per unit cell.

To also account for a smaller hydrogen load corresponding to a lower H chemical potential, a structure with single passivated surface metal ions and one hydrogen per anion is considered (Figure 1, lower path). This “H-single” passivation has 8 H atoms (4 H_2_ molecules) per unit cell. According to further test calculations, other passivation options including, for example, only covering metal ions or anions with hydrogen are energetically highly unfavored and will thus not be considered.

## Filtering for Stable Systems

**Figure 2:**
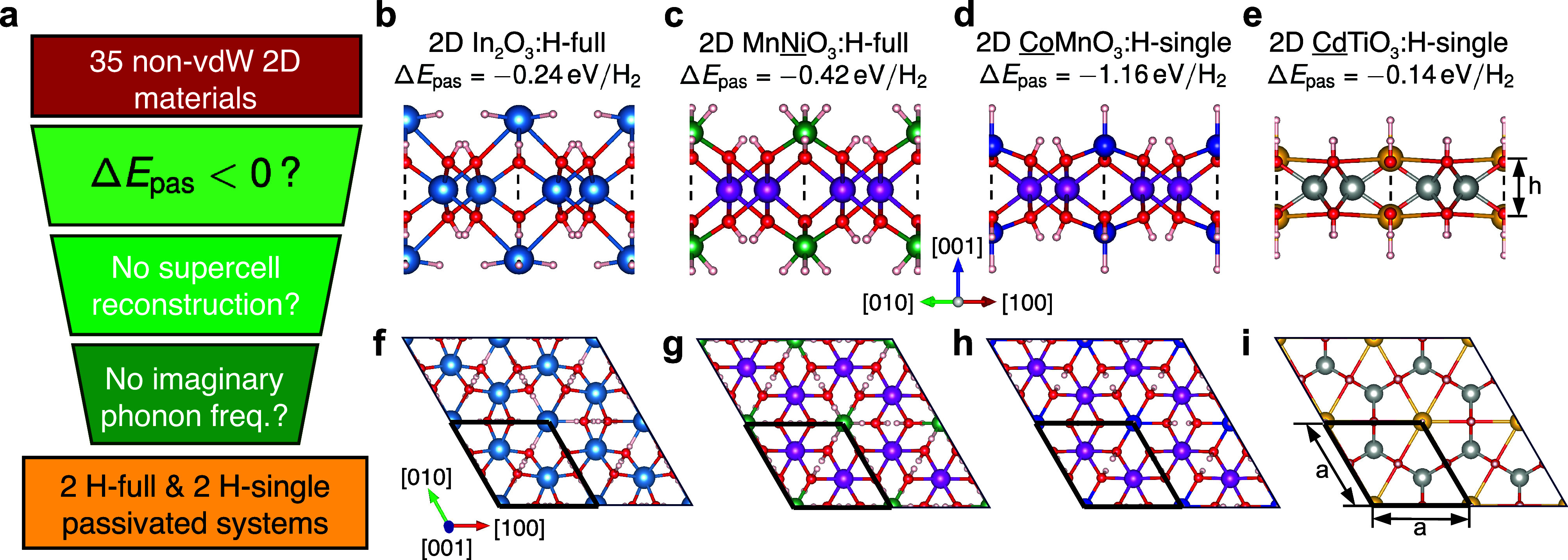
Non-vdW 2D material passivation. (a) Schematic workflow to identify stable passivated non-vdW 2D systems. (b–e) Side views of the relaxed structures of passivated candidates with their respective passivation types and energies indicated. The slab thickness *h*, measured between the outer cations, is represented in (e). For ternaries, the terminating elements of the pristine slabs (outer ions) are underlined in the formula to clearly indicate the passivated cation species. Periodic repetitions of the structure are separated by dashed vertical lines. The compass indicates crystal directions for all side views. (f–i) Top views of 2 × 2 supercells of the relaxed structures. The in-plane lattice constant *a* is indicated in (i). The area in the black dashed frames corresponds to the respective primitive unit cell. The compass indicates the crystal directions for all top views. Colors: H, off-white; O, red; Ni, green; Mn, purple; Co, dark blue; In, light blue; Ti, gray; Cd, yellow.

Figure 2a shows the filtering scheme to determine energetically favored and dynamically stable passivated non-vdW 2D systems. The first criterion for favorable hydrogenation is whether the system is lower in energy than its constituents—the pristine slab and free H_2_ molecules—i.e., whether the passivation energy Δ*E*_pas_ < 0. This is fulfilled for four H-full (Rh_2_O_3_, In_2_O_3_, CoMnO_3_, and MnNiO_3_) and nine H-single (Rh_2_O_3_, In_2_O_3_, AgBiO_3_, CdTiO_3_, CoMnO_3_, MnNiO_3_, CuVO_3_, SnZnO_3_, and BiNaO_3_) candidates.

To exclude systems susceptible to longer-range reconstructions, 2 × 2 in-plane supercells are generated from the relaxed geometries with the atomic coordinates randomized (Gaussian distribution, standard deviation 50 mÅ)^53^ and reoptimized. Out of the 13 candidates, eight lower their energies during supercell relaxation relative to the primitive cell. Only the remaining three H-full (Rh_2_O_3_, In_2_O_3_, and MnNiO_3_) and two H-single (CoMnO_3_ and CdTiO_3_) passivated systems are further considered. To give an impression of the energy lowering due to reconstruction, for the H:single cases, the progression of the supercell energy with respect to the primitive cell is depicted in section I in the Supporting Information.

To verify whether the remaining five structures are indeed local minima in the potential energy landscape, phonon dispersions are computed^54^ (filtering step three). The two H-full (H-single) passivated candidates In_2_O_3_ and MnNiO_3_ (CoMnO_3_ and CdTiO_3_) do not show imaginary frequencies and are thus vibrationally stable. The phonon dispersions and density of states (DOS) for all five systems can be found in section II in the Supporting Information. Although during the full stability screening the amount of passivated candidates reduces significantly, we believe that the systems that have been filtered out but still exhibit unsaturated broken bonds at their surfaces might be successfully passivated by other groups such as OH as already indicated for 2D magnetite^23^ offering also a versatile playground for their property control.

Side/top views of the relaxed structures of the final four systems are depicted in Figure 2b–e/f–i. The passivation energies are also included and vary between −0.14 eV/H_2_ for the CdTiO_3_:H-single and −1.16 eV/H_2_ for the CoMnO_3_:H-single. All values are much larger than the room temperature thermal energy of ∼25 meV indicating that these systems remain passivated under ambient conditions. At the same time, these moderate Δ*E*_pas_ values reveal that the hydrogenation can be reversible as found for graphene^52^ furthering the value of the associated magnetic state control.

We also estimate the energy penalty due to entropy loss −*T*Δ*S* upon hydrogen coverage. As an upper bound, one can assume that the entropy of free H_2_ molecules is completely lost upon passivation (neglecting vibrational entropy effects). If one takes the tabulated room temperature (298.15 K) entropy of H_2_ gas of 130.680 J/(K mol) from the NIST-JANAF thermochemical tables,^55^ −*T*Δ*S* amounts to ∼0.40 eV/H_2_, thus overcompensating the passivation energy of CdTiO_3_:H-single and In_2_O_3_:H-full. When repeating the same estimate at 100 K, only ∼0.10 eV/H_2_ is obtained, indicating the feasibility of passivating these systems at diminished temperatures.

## Passivated Structures

The passivation leads to pronounced structural changes relative to those of the pristine slab. The thickness *h*, measured between the two outer cations for comparability to the pristine case, is indicated in Figure 2e, whereas the in-plane lattice constant *a* is visualized in Figure 2i. The modifications with respect to the pristine compounds are summarized in Table 1. The relative changes are defined as Δ*h* = (*h*_passivated_ – *h*_pristine_)/*h*_pristine_ and Δ*a* = (*a*_passivated_ – *a*_pristine_)/*a*_pristine_, while the absolute change is the difference between the passivated and pristine values.

**Table 1:** Structural Changes—Relative/Absolute Changes of Slab Thickness Δ*h* and In-Plane Lattice Constant Δ*a* from Pristine to Passivated Systems

System	Δ*h* (%/Å)	Δ*a* (%/Å)
In_2_O_3_:H-full	+106/+3.26	+2/+0.14
MnNiO_3_:H-full	+53/+1.61	+6/+0.32
CoMnO_3_:H-single	+26/+0.78	+12/+0.66
CdTiO_3_:H-single	–18/–0.59	+12/+0.59

In general, the materials exhibit a thickness increase upon passivation, which is particularly pronounced for the H-full scenario reaching 106% for In_2_O_3_. When initially going from the as sliced bulk to the optimized pristine 2D systems, a thickness reduction on the order of 20–40% relative to the bulk value was observed due to strong surface relaxations.^39,40^ The hydrogens saturate the surface dangling bonds created upon exfoliation, thereby counteracting this thickness reduction and even overcompensating it for the H-full systems. CdTiO_3_:H-single is a special case for which the unique structural changes upon passivation were verified carefully by obtaining the final structure from different starting geometries. The oxygen atoms rearrange more strongly such that they end up in the same in-plane position at the top and bottom sides of the sheet (Figure 2e and i). To compensate for the stretched Cd–O bonds, the CdH group moves inward and thus reduces the slab thickness.

All systems exhibit a moderate increase of the in-plane lattice constants Δ*a* up to slightly above 10% for the H-single systems. This can be attributed to weaker in-plane bonds between the surface cations and O to compensate for the additional out-of-plane bonding with the passivating hydrogen leading to a slight stretching of the cells.

We focus in the following on how passivation tunes the magnetic properties. A comparison of the electronic properties of the pristine and passivated materials for all systems displaying, for instance, a modification of the band gaps is discussed in section III in the Supporting Information.

## Magnetism

**Figure 3:**
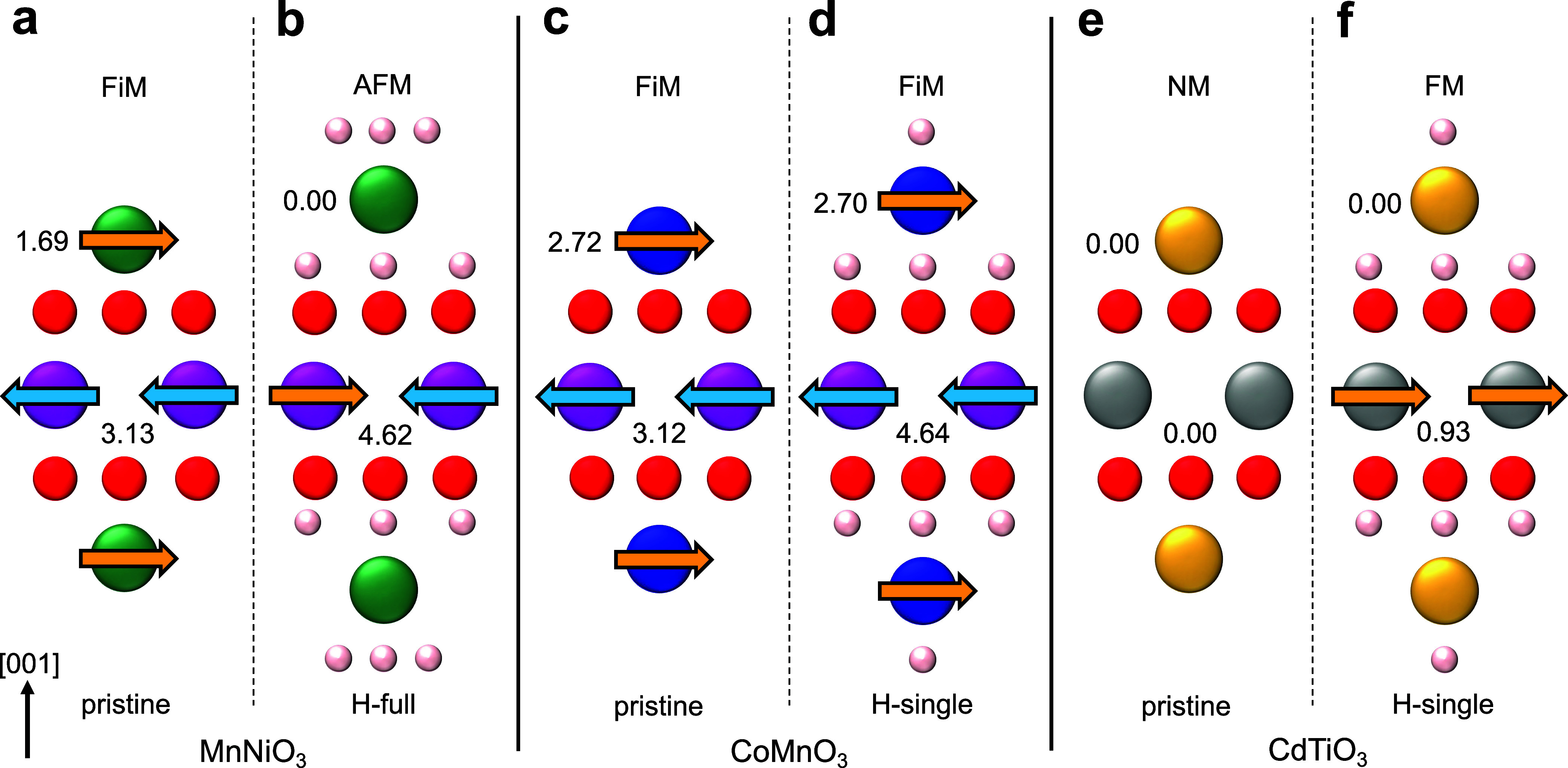
Magnetic configurations. Schematic side views of the magnetic configurations of pristine (a, c, e) and passivated (b, d, f) 2D systems with magnetic moments specified in μ_B_ for the selected cations. The compositions for which the specific configuration applies are indicated at the bottom.

As outlined in Figure 3, the magnetic states of the three systems critically depend on whether they are passivated (In_2_O_3_ remains diamagnetic upon hydrogenation). We focus in the following on the lowest energy magnetic states, while, for each system, a large amount of possible magnetic configurations has been checked as detailed in the Methods section. MnNiO_3_ displays a switch from a ferrimagnetic (FiM) configuration where the Mn moments of ∼3 μ_B_ are aligned antiparallel to the surface Ni ones of ∼1.7 μ_B_ to an antiferromagnetic (AFM) state with enhanced Mn moments of ∼4.6 μ_B_ and suppressed spin on Ni. This behavior can be analyzed by computed Bader charges. While these Bader values do not fully coincide with the formal oxidation states due to the incomplete ionization of the species, they provide a powerful way to analyze trends and changes of the oxidation states that by themselves determine the magnetic moments. For the pristine slab, the Mn and Ni charges of 1.88 and 1.21 *e*, respectively, correspond well to the values of 1.89 and 1.21 *e* for bulk MnO_2_ and NiO as determined within the AFLOW-CCE method with similar computational parameters.^56,57^ In the passivated case, electronic charge is transferred to these positively charged Mn and Ni sites, reducing the values to 1.48 and 0.66 *e*. This allows us to identify a switch of the Mn state from +4 to +2 since the value for the passivated system is closer to the 1.37 *e* known from MnO compared to the ∼1.7 *e* of Mn_2_O_3_ from the AFLOW database.^49,50^ This oxidation state fits well to the Mn moment from above close to 5 μ_B_ for the *d*^5^ electron system Mn^2+^. The strong charge transfer to Ni is indicative of a state moving toward Ni^0^ as the 0.66 *e* are much smaller than the Bader charge of ∼1.21 *e* of the lowest common oxidation state Ni^2+^.^56,57^

For CoMnO_3_, both the pristine and passivated structures show an FiM state albeit with the Mn moment again increased from 3.12 to 4.64 μ_B_. The change in the Bader charges from 1.87 to 1.50 *e* corroborates again the switch from Mn^4+^ to Mn^2+^. Also a mild charge transfer to the surface Co^2+^ of ∼0.22 *e* (from ∼1.26 to ∼1.04 *e*) is found, which, however, does not affect its magnetic moment.

Of particular interest is CdTiO_3_ (Figure 3e,f) where an onset of a ferromagnetic (FM) state with Ti moments close to 1 μ_B_ from the nonmagnetic (NM) pristine sheet is observed. Several AFM configurations within a 2 × 2 in-plane supercell have also been computed and are all higher in energy than the FM state as detailed in section IV.A in the Supporting Information. The change of the Ti charges from ∼2.37 to ∼2.12 *e* corresponds well to the data for Ti^4+^ and Ti^3+^ of ∼2.40 *e* and ∼2.02 *e* from bulk TiO_2_ (rutile) and Ti_2_O_3_.^56,57^ Like the Ni case above, the strong charge transfer of ∼0.8 *e* (from ∼1.20 to ∼0.39 *e*) to Cd^2+^ reveals a state getting close to Cd^0^ upon passivation with no local moment.

**Figure 4:**
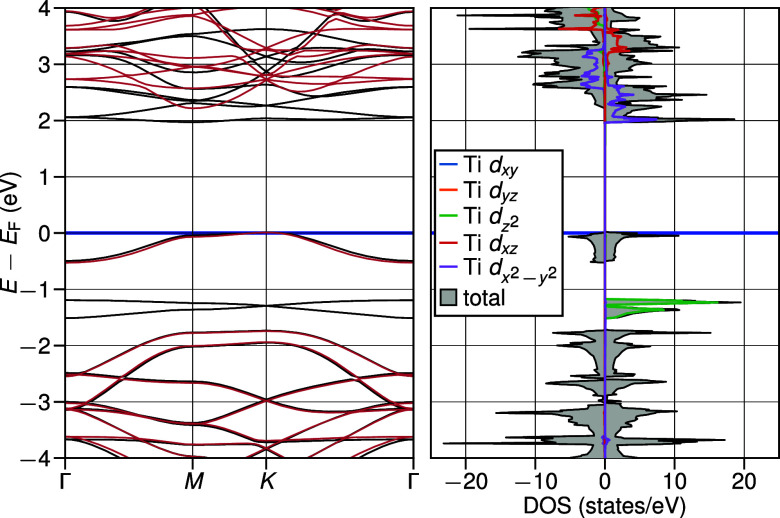
Band structure and projected DOS for CdTiO_3_:H-single. Band structure as well as total DOS of CdTiO_3_:H-single and its projection onto the different Ti *d* states. In the band structure, majority spin bands are indicated in black, whereas minority spin bands are in red. Following the AFLOW standard,^58^ the energies are aligned at the Fermi energy *E*_F_ at the top of the valence band. In the DOS, the majority spin states are indicated by positive values, while the minority spin states are negative.

The emergence of the magnetic moment at Ti can be traced in detail in the band structure and the atom and orbital projected DOS for the passivated system in Figure 4. At around −1.5 eV in the majority spin channel, the occupation of a Ti 3*d*_*z*^2^_ state is clearly seen, whereas the remaining DOS of the occupied states shows no significant spin asymmetry.

The magnetic behavior of this system is further characterized by its magnetic anisotropy, referring to the relative energy of all moments aligning along different spatial directions. Taking into account spin–orbit coupling, we find this energy difference between all moments pointing out-of-plane along [001] vs the in-plane [120] easy axis (direction perpendicular to [100]) to be ∼74 μeV/Ti signifying preferential in-plane alignment. Within the 2D plane, only a very minor variation of ∼4 μeV/Ti between [100] vs [120] is present. A detailed visualization of the angular dependence of the in-plane anisotropy is available in section IV.B in the Supporting Information.

Based on the energy difference of ∼2.5 meV/formula unit for the FM and AFM magnetic configurations in the primitive unit cell for CdTiO_3_:H-single, the Ti–Ti magnetic exchange coupling constant *J*_Ti–Ti_ and associated Curie temperature can be estimated. In analogy to refs (40 and 59−61), the energy difference can be mapped onto a nearest neighbor Heisenberg model *H* = −∑_⟨*i*>*j*⟩_ *J*_*ij*_**m**_*i*_**m**_*j*_, where *J*_*ij*_ is the magnetic exchange coupling constant between ions *i* and *j* and **m**_*i*_ and **m**_*j*_ stand for the magnetic moments at these sites. The resulting coupling constant amounts to *J*_Ti–Ti_ ∼ 0.82 meV. The mean-field Curie temperature *T*_C_^MF^ can now be estimated from *k*_B_*T*_C_^MF^ = *J*_0_/3 as explained in ref (62) (*k*_B_ is Boltzmann’s constant) by summing all the coupling constants of one Ti ion as *J*_0_ = 3*J*_Ti–Ti_. Our coupling constants are defined as twice the value in ref (62). We thus arrive at *T*_C_^MF^ ∼ 10 K. However, it is well-known that mean-field theory can slightly overestimate Curie temperatures.^62^

**Figure 5:**
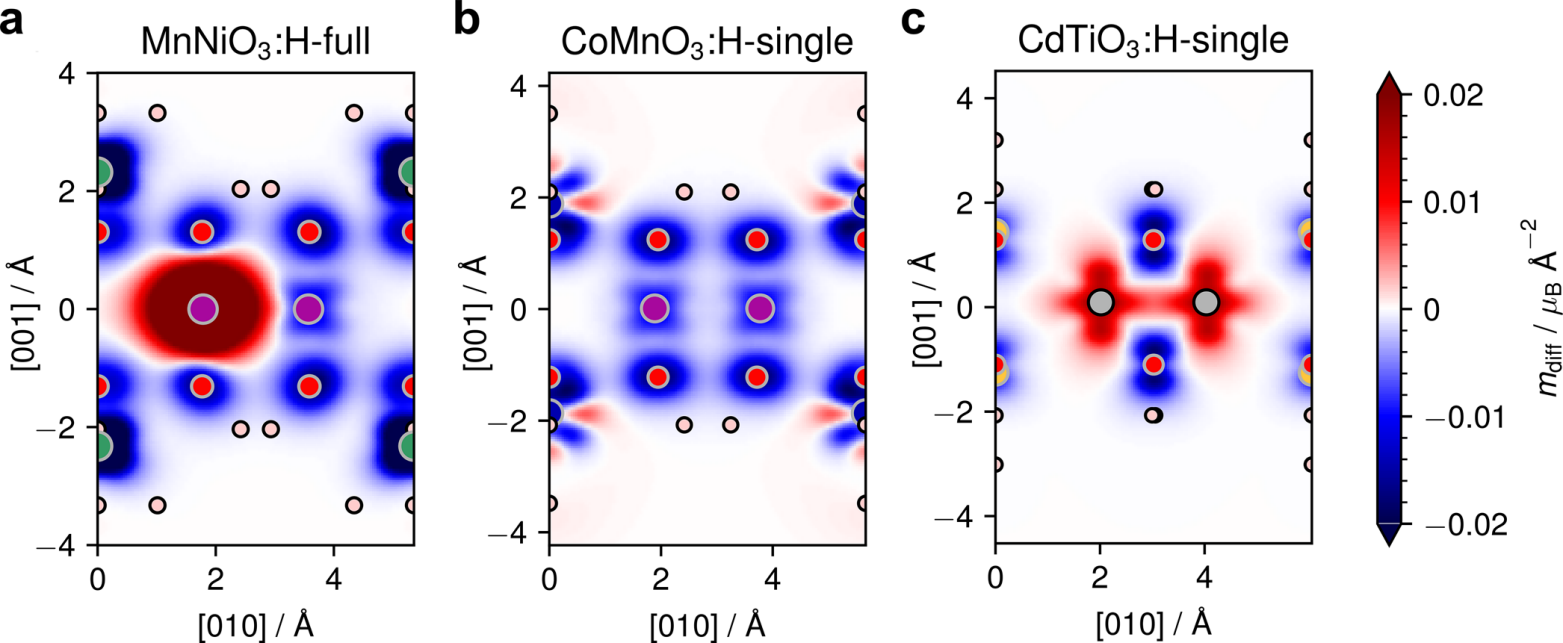
Magnetization density difference. Magnetization density difference (side view) between passivated and pristine MnNiO_3_ (a), CoMnO_3_ (b), and CdTiO_3_ (c). Red (blue) indicates a positive (negative) change in the magnetization density due to hydrogenation. The small circles represent atoms with the same color code as above. Each figure shows one unit cell.

In Figure 5, the strong change of the magnetic states upon passivation is further analyzed in detail around the cations for all cases in real-space by magnetization density difference plots (integrated over [100]). The magnetization density of a system is defined as the difference between spin up and spin down electron densities weighted by the Bohr magneton μ_B_: *m*(**r**) = μ_B_(*n*_*↑*_(**r**) – *n*_*↓*_(**r**)). As a measure of change, the magnetization density difference between the passivated and pristine systems can be computed as *m*_diff_(**r**) = *m*_passivated_(**r**) – *m*_pristine_^static^(**r**). In order to study the change of the magnetic configuration of each transition metal center at a fixed position, the latter quantity is evaluated for a strained pristine structure by a single static electronic calculation of the passivated geometry with the hydrogens removed. In the expression, the subtraction of the magnetization density of free hydrogen atoms at the positions of the passivated system is omitted. These have a moment of 1 μ_B_ each, but we are interested in the magnetic transition upon passivation from the pristine to the passivated 2D system which would be heavily obscured by the (artificial) magnetization change at the hydrogens.

For MnNiO_3_:H-full, Figure 5a depicts a very strong positive change at the left Mn associated with the flipping and enhancement of the moment at this center upon passivation. The right Mn only shows a slight negative change due to the increase of the moment without flip. The data thus signifies the change of the magnetic symmetry between these moments from a mirror relation in the pristine to an inversion in the hydrogenated case. Also the strong moment reduction around the surface Ni upon passivation is evident from the dark blue region.

The *m*_diff_ of CoMnO_3_:H-single (Figure 5b) reveals the expected slight negative change at the central Mn due to the moment enhancement in the spin down direction. For the surface Co, both blue and red regions indicate a balance between magnetization gains and losses fitting well with the above finding of a roughly constant moment.

The dominant feature for the CdTiO_3_:H-single case (Figure 5c) is the strong magnetization gain at the two central Ti ions in the 3*d*_*z*^2^_ orbital shape in nice correspondence to the above DOS analysis confirming the onset of the FM state upon passivation. Note that, in all plots, a substantial negative magnetization change at the oxygens is visible. This is due to the fact that, in the strained pristine geometry deduced from the hydrogenated system, these ions carry a small moment of ∼0.3 to 0.5 μ_B_ which disappears upon passivation. The qualitative analysis of the magnetization change at the transition metal centers is, however, not impacted by this effect.

## Conclusions

We have demonstrated control of the magnetic state of transition metal based non-vdW 2D materials by surface hydrogenation utilizing data-mining and autonomous density functional theory calculations. The modification of the properties was analyzed in detail based on magnetic moments, Bader charges, and magnetization density differences. For 2D MnNiO_3_, hydrogenation induces a transition from a FiM to an AFM state with enhanced Mn moments and a switched local spin symmetry. In the case of 2D CoMnO_3_, the FiM state is preserved but the Mn moments are again enhanced. For CdTiO_3_, the onset of ferromagnetism upon passivation is revealed. Our findings thus corroborate that hydrogenation also complemented by other types of surface passivation in general can be a versatile and powerful tool to further magnetically “activate” these novel non-vdW 2D and likely other nanoscale systems.

## Methods

The passivation energy Δ*E*_pas_ is computed as the energy difference between the relaxed passivated system *E*_passivated_, the pristine non-vdW 2D material *E*_pristine_, and free H_2_ molecules *E*_H_2__ (the standard thermodynamic reference phase for hydrogen), normalized per H_2_
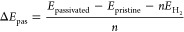
1where *n* = 4/6 is the number of hydrogen molecules added to the system for the respective passivation type.

The initial passivated structures are generated from the pristine geometries with hydrogen bond lengths estimated using the respective covalence radii. A vacuum of at least 20 Å separating periodic images of the slab in the [001] direction is inserted before relaxation. Both the ionic positions and the cell shape are allowed to relax, unless stated otherwise.

The calculations for the exchange-correlation functional PBE+*U*^63−66^ are performed with AFLOW^49,50^ and the Vienna *Ab-initio* Simulation Package (VASP)^67−69^ with settings (including DFT+*U* method and values) according to the AFLOW standard^58^ and the internal VASP precision set to ACCURATE. Calculations for slabs are performed on a Γ-centered 10 × 10 × 1 *k*-point grid, and the phonon dispersions are computed with AFLOW-APL^54^ by constructing 3 × 3 (H-full) or 4 × 4 (H-single) in-plane supercells (containing 198 and 288 atoms, respectively) without polar corrections.

The candidate systems containing potentially magnetic elements such as Ti, V, Cr, Mn, Fe, Co, Ni, and Rh are rigorously checked for magnetism using the algorithm developed within the coordination corrected enthalpies (CCE) method,^56^ i.e., investigating the NM and all possible FM and AFM configurations in the structural unit cell for five different sizes of induced magnetic moments each. The analysis is only applied to other systems when the standard workflow of AFLOW^58^ results in finite magnetic moments after the relaxation. For CdTiO_3_:H-single, further longer-range AFM configurations displayed in section IV in the Supporting Information are also considered. In each case, the lowest energy magnetic state is used for further calculations. The reference energy *E*_H_2__ was computed in a 10 Å × 10 Å × 10 Å cell using a Γ-only *k*-point sampling while relaxing the H–H bond length.

The magnetic anisotropy energies are calculated in a static self-consistent run along the different (Cartesian) spatial directions by taking into account spin–orbit coupling, switching off symmetry (ISYM = −1), and restoring complete lattice symmetry (GGA_COMPAT = .FALSE.). The results were carefully checked for convergence with respect to the number of *k*-points and bands within the calculation with the final values obtained with a Γ-centered 27 × 27 × 1 *k*-point grid and 152 bands.

## Data Availability

The primary research data of this study are available from the Rossendorf Data Repository (RODARE) via 10.14278/rodare.2496.

## Supporting Information Available

Energy progression during supercell relaxation for H-single systems, phonon dispersions and DOS of passivated systems, comparison of electronic band structures and DOS of pristine and passivated systems, AFM configurations for CdTiO_3_:H-single, and angular dependence of the in-plane magnetic anisotropy (PDF)
