# Supplementary material for: Magnetic State Control of Non-van der Waals 2D Materials by Hydrogenation
Source: Nano Lett. 2024 Mar 6;24(13):3874–81. doi: 10.1021/acs.nanolett.3c04777 (PMC10996018; doi:10.1021/acs.nanolett.3c04777)
Supplement: Supplementary file 1 — nl3c04777_si_001.pdf [file nl3c04777_si_001.pdf]

Magnetic State Control of Non-van der Waals 2D Materials by Hydrogenation Supporting Information

Tom Barnowsky,[1,2] Stefano Curtarolo,[3,4] Arkady V. Krasheninnikov,[2] Thomas Heine,[1,5] and Rico Friedrich[1,2,3,*]

[1] *Theoretical Chemistry, Technische Universität Dresden, 01062 Dresden, Germany*
[2] *Institute of Ion Beam Physics and Materials Research, Helmholtz-Zentrum Dresden-Rossendorf, 01328 Dresden, Germany*
[3] *Center for Extreme Materials, Duke University, Durham, NC 27708, USA*
[4] *Materials Science, Electrical Engineering, and Physics, Duke University, Durham, NC 27708, USA*
[5] *Center for Advanced Systems Understanding (CASUS), Helmholtz-Zentrum Dresden-Rossendorf, 02826 Görlitz, Germany*

(Dated: February 14, 2024)

I. Energy progression during supercell reconstruction

Fig. S1 shows the progression of the energy during the relaxation of the 2×2 supercell ($E_{2\times2}$) relative to the energy of the relaxed 1×1 cell ($4E_{1\times1}$). For all H-single passivated systems except $CdTiO_3$:H-single and $CoMnO_3$:H-single, the geometry reconstructs by lowering its energy below the one of the optimized 1×1 cell. These cells are thus considered unstable with respect to supercell reconstruction. Discontinuities in the energy as observable for $MnNiO_3$:H-single around relaxation step 130 and 180, are due to different magnetic solutions found for single magnetic ions during these optimization steps.

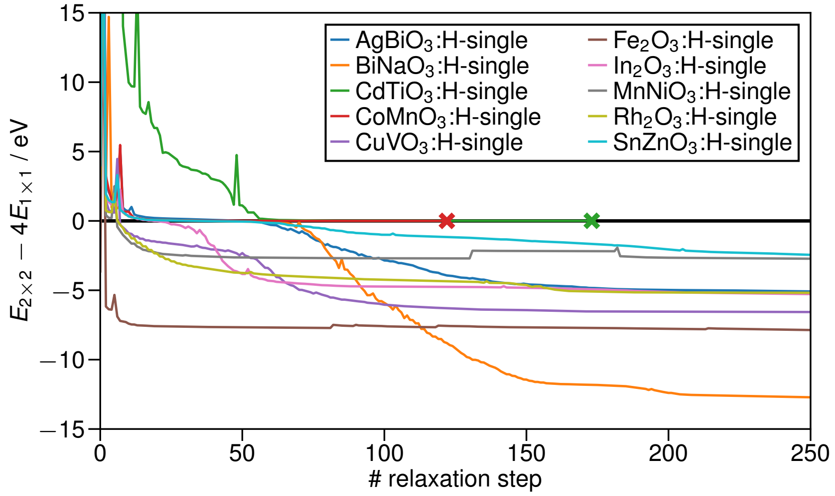

FIG. S1. **Energy progression during supercell reconstruction.** Energy progression during optimization of the randomized 2×2 supercells of the H-single passivated systems. If the required accuracy of total energy change of 10^{-5} eV per cell between subsequent structures is reached, the relaxation ends, which is marked with an $\times$ in the color of the respective curve.

* r.friedrich@hzdr.de

II. Phonon dispersion curves

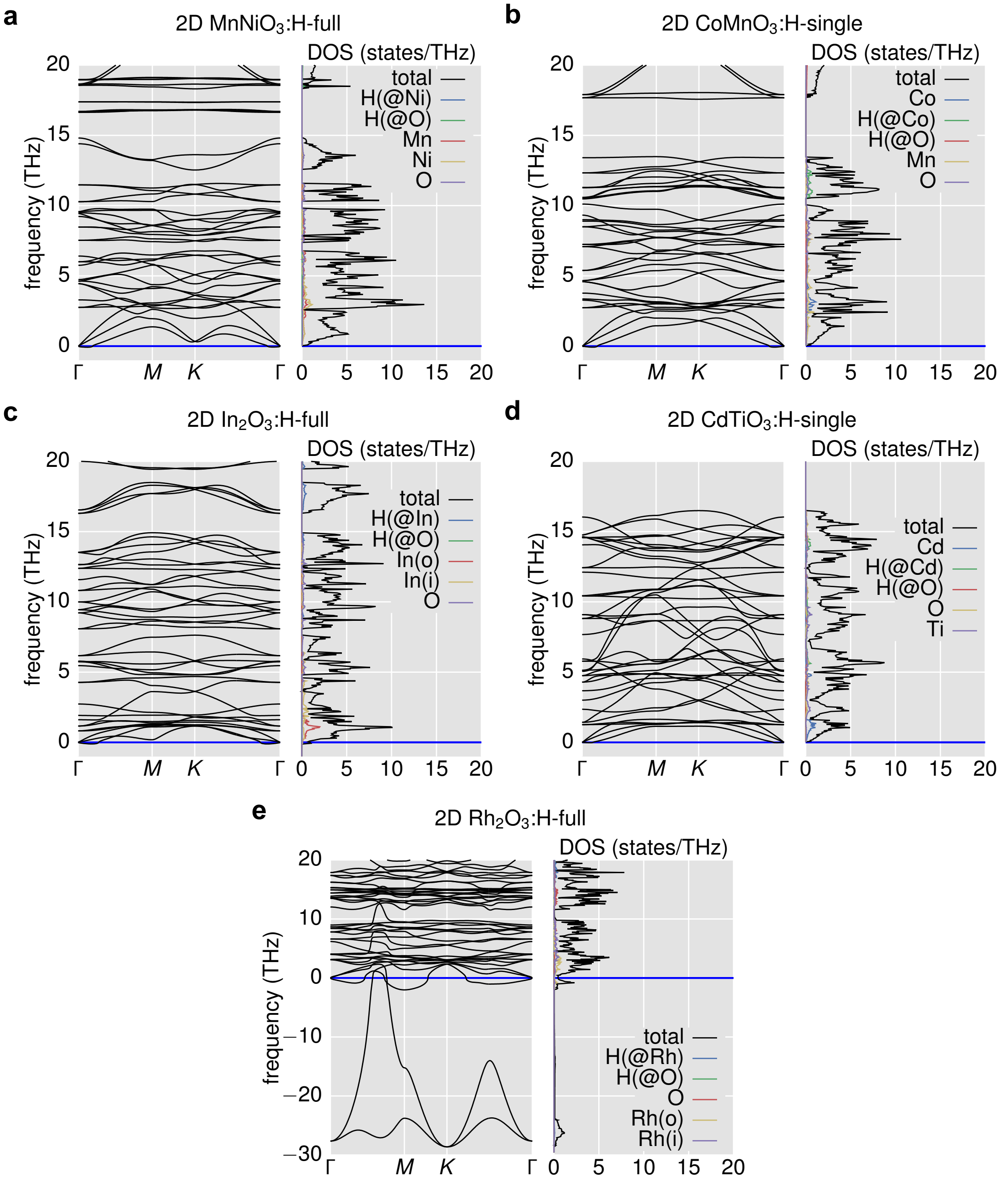

FIG. S2. **Phonon dispersions.** Phonon dispersion curves and density of states (DOS) for the passivated candidates (**a**–**e**) after the supercell reconstruction filter. Contributions by atoms at non-equivalent sites, namely inner and outer cations (labeled (i) and (o) if occupied by the same species for binaries), anions, and hydrogen (labeled (@X) when attached to site X), are indicated in the DOS. As pointed out in Ref. [1], a small imaginary dip close to Γ is sensitive to numerical details and does not indicate instabilities.

III. Electronic properties

A comparison of the bandstructures of the pristine and passivated 2D systems for each compound is depicted in Figs. S3-S6. For In_2O_3/$MnNiO_3$/$CoMnO_3$, the hydrogen coverage leads to a significant opening of the band gaps from 1.29 eV/1.79 eV/1.29 eV in the pristine to 2.98 eV/2.39 eV/2.35 eV in the passivated case. This opening of the gaps can be understood from the enhanced band splitting upon forming additional bonds to the surface hydrogen and the passivation of surface states originating from exfoliation [2]. $CdTiO_3$ is again a special case, since the band gap decreases from 2.75 eV to 1.96 eV upon H-single passivation. This can be correlated with the unique structural changes since the strongly elongated bonds in this system give rise to a reduced band splitting.

Also, the change in magnetic state is visible in the band structures. While In_2O_3 remains non-magnetic when passivated, $MnNiO_3$ switches from ferrimagnetic to antiferromagnetic upon H-full coverage. As such, the band structure of the passivated system depicts no net spin polarization. For $CoMnO_3$, the change of the size of the magnetic moments in the ferrimagnetic state due to H-single passivation goes along with a change in the relative spin character of the valence band top and conduction band bottom. Although for the pristine system, the states are both derived from minority spin, for the H-single covered compound, only the valence band top is minority spin derived whereas the conduction band bottom originates from majority spin states. In case of $CdTiO_3$:H-single, the initialization of the ferromagnetism upon passivation causes the states at the gap to both have minority spin character.

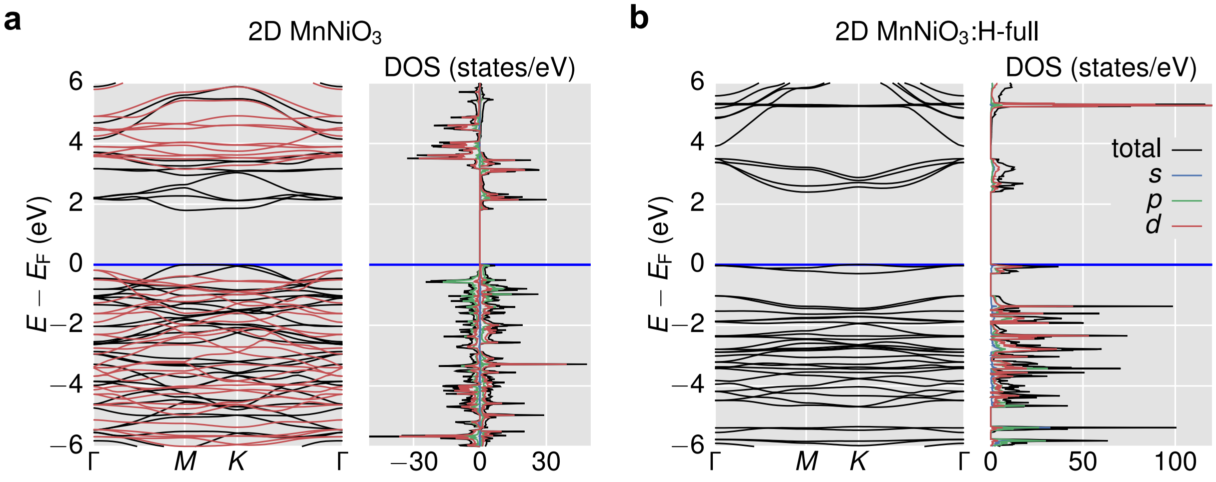

FIG. S3. Bandstructure and density of states for pristine (**a**) and passivated (**b**) $MnNiO_3$. Following the AFLOW standard [3], the energies are aligned at the respective Fermi energy E_F at the top of the valence band. For the spin polarized bandstructure, majority spin bands (positive DOS) are indicated in black, while minority spin bands (negative DOS) are in red.

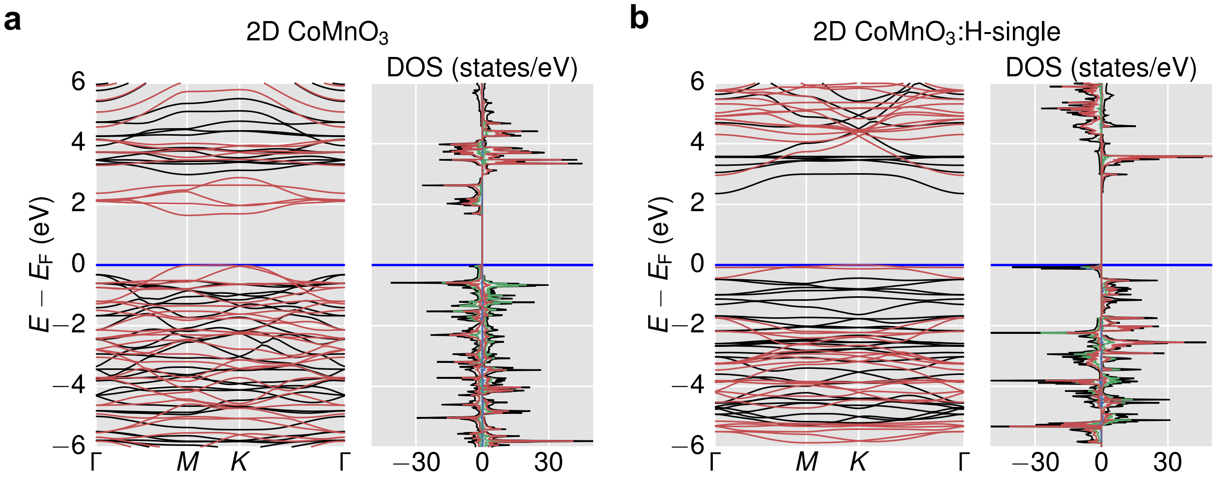

FIG. S4. Bandstructure and density of states for pristine (**a**) and passivated (**b**) $CoMnO_3$.

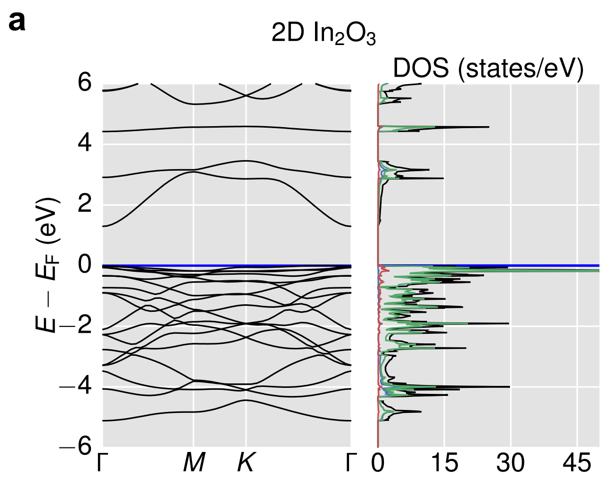

FIG. S5. Bandstructure and density of states for pristine (**a**) and passivated (**b**) In_2O_3.

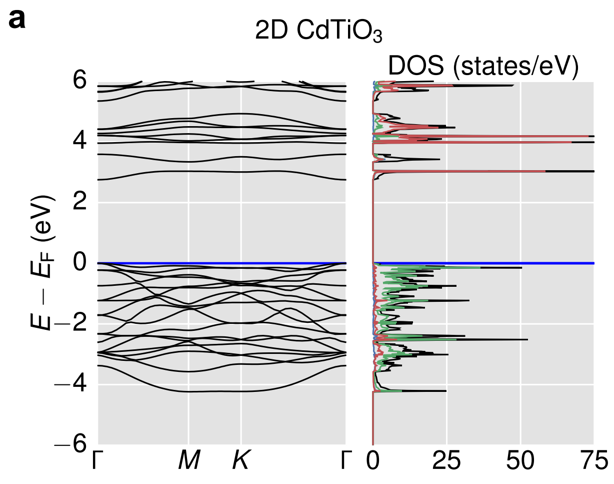

FIG. S6. Bandstructure and density of states for pristine (**a**) and passivated (**b**) $CdTiO_3$.

IV. Magnetic properties of $CdTiO_3$:H-single

A. Antiferromagnetic configurations

As the magnetic unit cell of a system can be in general larger than the structural one, we sampled the magnetic configuration space by four AFM states for $CdTiO_3$:H-single to verify the energetic preference of the FM state. Fig. S7 depicts 2×2 supercells with the respective magnetic configuration of the Ti moments indicated by the color pattern. The first AFM configuration can still be represented in the structural unit cell and has been used to compute the coupling constants, but all other states show a longer-range AFM ordering requiring the 2×2 supercell construction. All states are higher in energy than the FM configuration, corroborating its energetic favorability.

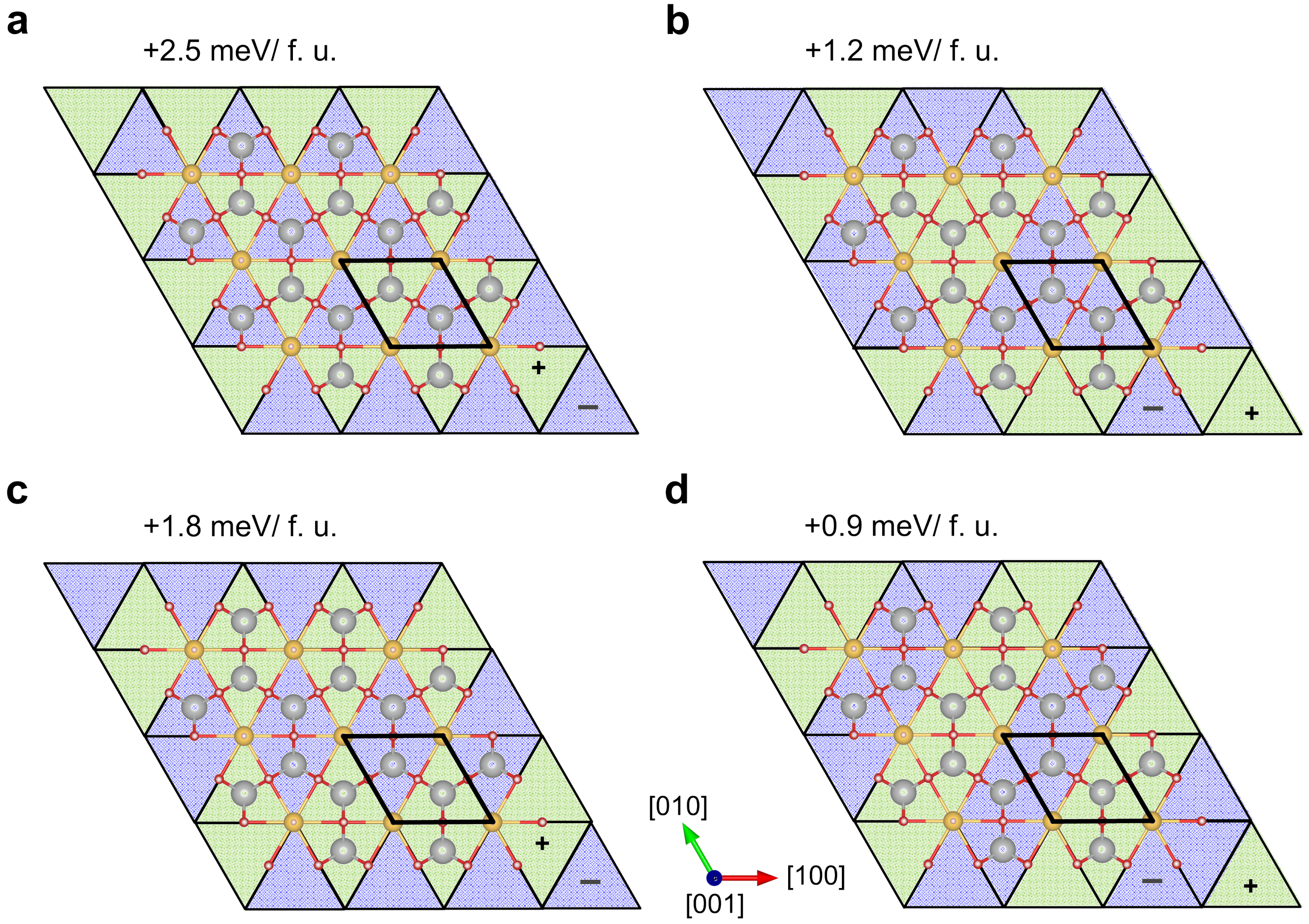

FIG. S7. **Antiferromagnetic configurations for $CdTiO_3$:H-single.** AFM configurations considered for $CdTiO_3$:H-single depicted in a 2 × 2 in-plane supercell. Primitive unit cell (indicated by the black frame) AFM configuration (**a**) and different longer-range AFM configurations (**b** – **d**). The respective energy difference to the FM state in meV/formula unit (f. u.) is indicated in each case. The green (blue) shaded regions around each Ti ion, also extending over the unit cell boundary for better visualization, represent upward,+ (downward,−) alignment of the respective magnetic moments.

B. In-plane magnetic anisotropy

To determine the energetically pereferred magnetization direction in the plane of the 2D slab, the in-plane magnetic anisotropy energy (MAE) was computed for several directions. Fig. S8 shows the energy differences arising when the magnetic moments of the Ti atoms are aligned in different in-plane directions. The energy minimum in [210] (equivalent to [120]) = 30° direction is clearly visible.

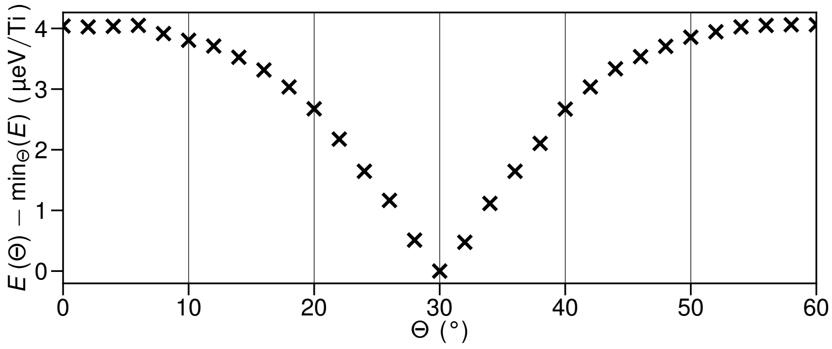

FIG. S8. **In-plane magnetic anisotropy energy for $CdTiO_3$:H-single.** In-plane sweep of the MAE for $CdTiO_3$:H-single over all unique in-plane angles in the unit cell (from [100] = 0° to [110] = 60°) in steps of 2°.

REFERENCES

[1] S. Radescu, D. Machon, and P. Mélinon, *Origin of dynamical instabilities in some simulated two-dimensional materials: GaSe as a case study*, Phys. Rev. Materials **3**, 074002 (2019).

[2] R. Friedrich, M. Ghorbani-Asl, S. Curtarolo, and A. V. Krasheninnikov, *Data-Driven Quest for Two-Dimensional Non-van der Waals Materials*, Nano Lett. **22**, 989–997 (2022).

[3] C. E. Calderon, J. J. Plata, C. Toher, C. Oses, O. Levy, M. Fornari, A. Natan, M. J. Mehl, G. L. W. Hart, M. Buongiorno Nardelli, and S. Curtarolo, *The AFLOW standard for high-throughput materials science calculations*, Comput. Mater. Sci. **108 Part A**, 233–238 (2015).
